# W. Patrick McCray 2020: *Making Art Work. How Cold War Engineers and Artists Forged a New Creative Culture*. Cambridge, MA: MIT Press, geb., 384 S., 52 Abb., 45 US $, ISBN: 9780262044257.

**DOI:** 10.1007/s00048-023-00368-9

**Published:** 2023-08-24

**Authors:** Vera Wolff

**Affiliations:** 1 documenta Institut, Kassel, Deutschland; 2 Universität Zürich, Zürich, Schweiz

Mitte der 1960er Jahre entstanden in den Zentren des militärisch-akademisch-industriellen Komplexes der USA Projekte und Institutionen, in denen Ingenieur*innen und Wissenschaftler*innen mit Künstler*innen zusammenarbeiten sollten. Zu den berühmtesten zählen das vom Los Angeles County Museum initiierte Art and Technology Program, das MIT Center for Advanced Visual Studies und die Experiments in Art and Technology (E.A.T.). Ihr Nachleben ist heute in unzähligen, der künstlerischen Forschung gewidmeten Programmen zu beobachten.

Der Wissenschaftshistoriker Patrick McCray interessiert sich für die Rolle der Ingenieur*innen und den Beitrag, den sie für die genannten Projekte geleistet haben. Sein Interesse entspricht einer allgemeinen Entwicklung. So ist beispielsweise in der Architekturgeschichte jüngst dazu übergegangen worden, nicht nur die Leistungen der Meisterarchitekt*innen zu beschreiben, sondern auch die Beiträge der vielen unbekannten Ingenieur*innen zu untersuchen. In Abgrenzung zu dem, was er als klassisch kunsthistorisches Vorgehen versteht, will McCray nicht von den aus der Kollaboration von Künstler*innen und Ingenieur*innen entstandenen Kunstwerken handeln, sondern von den Prozessen der Zusammenarbeit. Seine Geschichte der Entstehung einer „neuen kreativen Kultur“ erzählt er gleichwohl hauptsächlich anhand von Biografien – darunter denen von so einflussreichen und außergewöhnlichen Figuren wie Frank J. Malina und Billy Klüver.

Malina war Direktor des Jet Propulsion Lab am Caltech, bevor er während der McCarthy-Zeit Schwierigkeiten wegen seiner Nähe zur Kommunistischen Partei der USA bekam. Später wurde er Chef der Abteilung für Scientific Research bei der UNESCO. 1968 gründete er die Zeitschrift *Leonardo*, die Künstlern Zugang zu Informationen über die neuesten technischen Entwicklungen und aktuelle wissenschaftliche Forschung bieten sollte und zu einem der wichtigsten Sprachrohre für die digitalen Künste wurde. Der Elektroingenieur Klüver war bei Bell Labs angestellt, wo Mitte der 1960er Jahre vierzig Prozent der Belegschaft an militärischen Projekten arbeitete, und 1967 Mitbegründer der gemeinnützigen Organisation Experiments in Art and Technology, die sich von der Kollaboration zwischen Künstler*innen, Wissenschaftler*innen und Ingenieur*innen nicht weniger als die Vermeidung einer befürchteten „Kulturrevolution“ versprach. Weder zu Malina noch zu Klüver existierten bisher ausführlichere Untersuchungen. Allein deswegen stellt McCrays Arbeit eine wichtige Ergänzung der vorhandenen Literatur dar.

Im Zuge der quellengesättigten Darstellung stellt sich jedoch der Eindruck ein, dass der Autor seine Protagonist*innen als Verkörperung der Lösung des von C. P. Snow nach dem Sputnikschock beschworenen Konflikts der „zwei Kulturen“ auffasst. McCray hebt die Innovativität seiner Figuren hervor. Die in den Quellen ubiquitär auftauchenden Aussagen zur Kreativität und zur Vergleichbarkeit von Kunst und Ingenieurswesen behandelt er aber nicht durchgängig als historische Behauptungen. Zwar skizziert er die Kontexte und Interessen, die solche Aussagen legitimieren sollten und die von der Zivilisierung der Kriegstechniken bis zur an den Standards der MINT-Fächer orientierten Reform der Universitäts- und Hochschulausbildung für das damals prophezeite „postindustrielle Zeitalter“ reichen. Dennoch wirkt es vielfach so, als ginge es McCray um eine erneute Behauptung des Kreativitätsparadigmas, das die digitale „Wissensgesellschaft“ unserer Tage aus der Zeit des Kalten Krieges geerbt hat.

Analog zu den interdisziplinär organisierten „Big Sciences“ des Kalten Krieges nennt McCray die aus der Zusammenarbeit von Künstler*innen, Ingenieur*innen und Wissenschaftler*innen hervorgegangenen Projekte „Big Art“. Die von ihm untersuchten Kollaborationsprojekte lösten intensive Debatten über die sozialen, politischen und ökonomischen Dimensionen von Wissenschaft und Technik aus und manche intendierten dies auch. Damit haben sie – trotz ihrer Emphase für Interdisziplinarität und Teamwork – weniger mit Atomphysik oder Raketenforschung gemein als mit den damals in kritischer Auseinandersetzung mit den „Big Sciences“ entstandenen Science and Technology Studies, die die gesamtgesellschaftlichen Dimensionen von Wissenschaft und Technik zu ihrem Gegenstand machten.

Mit *Making Art Work* verknüpft McCray den Anspruch, die Art und Weise, wie Kunstgeschichte geschrieben wird, zu revolutionieren, indem er die Perspektive von den Werken auf die Arbeitsprozesse verlagert. Leider behandelt er die in den Projekten zum Einsatz gekommenen Techniken und Werkzeuge aus den Arsenalen des Kalten Krieges am Ende kaum. Daran ändert auch das suggestive Verfahren, seine Kapitel mit den Namen technischer Gerätschaften zu überschreiben („preamplifier“, „heterodyne“ oder „transducer“) nichts, denn diese dienen vor allem der metaphorischen Charakterisierung der Projekte und Protagonist*innen. Merkwürdigerweise erinnert McCrays primär auf Biografien und Projektgeschichten abgestellte Darstellungsweise ausgerechnet an das älteste Modell kunsthistorischen Erzählens: Giorgio Vasaris Mitte des 16. Jahrhunderts verfassten *Vite* der Künstler*innen der Renaissance.

